# Dynamics of CRISPR Loci in Microevolutionary Process of *Yersinia pestis* Strains

**DOI:** 10.1371/journal.pone.0108353

**Published:** 2014-09-29

**Authors:** Maria Paloma S. Barros, Camila T. França, Rosanny Holanda F. B. Lins, Milena Danda V. Santos, Ednaldo J. Silva, Maria Betânia M. Oliveira, Vladimir M. Silveira-Filho, Antônio M. Rezende, Valdir Q. Balbino, Tereza Cristina Leal-Balbino

**Affiliations:** 1 Departamento de Microbiologia, Centro de Pesquisas Aggeu Magalhães (CPqAM/Fiocruz), Recife, PE, Brasil; 2 Departamento de Genética, Universidade Federal de Pernambuco (UFPE), Recife, PE, Brasil; 3 Departamento de Bioquímica, Universidade Federal de Pernambuco (UFPE), Recife, PE, Brasil; 4 Departamento de Biologia, Universidade de Pernambuco (UPE), Garanhuns, PE, Brasil; University of Helsinki, Finland

## Abstract

The potential use of CRISPR loci genotyping to elucidate population dynamics and microevolution of 146 *Yersinia pestis* strains from different biovars and locations was investigated in this work. The majority of strains from the Orientalis biovar presented specific spacer arrays, allowing for the establishment of a CRISPR signature for their respective isolates. Twenty-one new spacers were found in the *Y. pestis* strains from plague foci in Brazil. Ninety-three (64%) strains were grouped in the G1 genotype, whereas the others were distributed in 35 genotypes. This study allowed observing a microevolutionary process in a group of *Y. pestis* isolated from Brazil. We also identified specific genotypes of *Y. pestis* that were important for the establishment of the bacteria in plague foci in Brazil. The data have provided supporting evidence for the diversity and dynamics of CRISPR loci present in the genome of *Y. pestis* strains from plague foci in Brazil.

## Introduction

The CRISPR loci (Clustered Regularly Interspaced Short Palindromic Repeats) consist of DNA repeats interspaced by non-repetitive elements or ‘spacers’ which are usually incorporated from foreign genetic elements (viruses or plasmids). These loci and Cas proteins (CRISPR associated protein) form an adaptive immune system that protect bacteria against invading phages and plasmids as well as participate in cellular regulatory mechanisms [1], [2].

The *Yersinia pestis* genome contains three CRISPR loci called YPa (YP1), YPb (YP2) and YPc (YP3) and, presently, 137 spacer sequences have been reported [3], [4], [5]. The distribution and spacers/spacer arrays in *Y. pestis* associate with plague foci, allowing the construction of a microevolutionary hypothesis for the isolates [4].

The current classification which reflects *Y. pestis* global diversity is based on SNP typing and also on different biochemical characteristics [5], [6], [7], [8]. Four biovars of *Y. pestis* (Antiqua, Medievalis, Orientalis, and Xilingolensis) were analyzed in this study. Our work explored the potential use of CRISPR loci genotyping for the study of 146 *Y. pestis* strains from different geographical sites. Finally we describe a new set of spacers, which contributed to the understanding of the microevolution of *Y. pestis* isolated from plague foci in Brazil. In addition, the data presented here are compatible with a single entry of *Y. pestis* in Brazil.

## Materials and Methods

### Bacterial strains and culture conditions

A total of 128 *Y. pestis* strains belonging to the *Yersinia* culture collection (FIOCRUZ – CYP) of the Centro de Pesquisas Aggeu Magalhães were selected for this study (Table S1). Biochemical analyses were performed during the isolation period and all of them were found to belong to the Orientalis biovar. Strains were isolated from humans, rodents, and fleas from 1966 to 1997 in 5 plague foci of Brazil. In addition, 18 strains from plague foci from Asia, Africa and America were included (Table S1). The cultures were maintained in peptone agar at 4°C, and were inoculated into BHI (Brain Heart Infusion Broth, HIMEDIA) at 28°C for 48 h. Each culture was plated in BAB (Blood Agar Base, HIMEDIA) and was incubated at 28°C for 48 h.

### DNA extraction and CRISPR loci amplification

Genomic DNA was extracted using the method described by Keim et al. [9], and adapted by Oliveira et al. [10]. PCR reactions were performed as described by Le Flèche et al. [11], and 3 CRISPR loci (YPa, YPb, YPc) were amplified with primer pairs described by Pourcel et al. [3].

### Amplicon purification and sequencing

PCR products were purified using the Purelink PCR Purification Kit (Invitrogen, Brazil) following the user’s manual. The purified products were sequenced on an Applied Biosystems 3100 automated DNA sequencer using the BigDye Kit (Applied Biosystems, Brazil).

### CRISPR loci analysis

Sequence assembly and editing were performed with the Seqman module of the DNAstar package (DNAstar Inc., Madison, Wis.), MEGA v. 5.0 [12] and Gene Runner 3.05 (Hastings Software, Inc). CRISPRFinder (available at http://crispr.u-psud.fr/) was used to confirm the identity of the sequences found. The spacer sequences were compared against the microbial genome database in GenBank (available at http://www.ncbi.nlm.nih.gov), using the sequence alignment tool BLAST to find similar sequences [13]. The spacer nomenclature used in this work for *Y. pestis* was based on the new CRISPR database generated by Mikael Skurnik for *Yersinia pseudotuberculosis* complex species. The CRISPR loci of 10 *Y. pestis* published genomes (A1122: NC_017168, Z176003: NC_014029, D106004: NC_017154, D182038: NC_017160, Harbin 35: NC_017265, Angola: NC_010159, Antiqua: NC_008150, Nepal516: NC_008149, 91001: NC_005810, Pestoides F: NC_009381) were amplified through *in silico* PCR software (available at http://insilico.ehu.es/PCR/).

### Genotype Network

In order to establish relationships among the genotypes found for *Y. pestis* using the CRISPR loci, the eBURST version 3.0 tool [14], [15] was employed. The rationale utilized in eBURST simplifies the problem of depicting the evolutionary relationship among closely related genotypes. Different from cluster diagrams, trees or dendrograms, it uses a simple but appropriate model of bacterial evolution in which an ancestral (or founding) genotype increases in frequency in the population, and while doing so, begins to diversify to produce a cluster of closely-related genotypes that are all descended from the founding genotype. This cluster of related genotypes is often referred to as a “clonal complex” [14], [15].

In order to run the eBURST, first a binary matrix was built, where 0 and 1 means absence or presence of a specific spacer sequence from one of the CRISPR loci, respectively. The matrix was divided into three sections corresponding to the three CRISPR loci that we found. Subsequently, profiles were built clustering identical spacer sequence arrays for each CRISPR locus. These profiles were combined into shorter genotypes for each sample and used as input for eBURST. A clonal complex was defined as a group of two or more independent genotypes that share identical profiles at a minimum of two loci with at least one other member of the group.

### Evolutionary analysis of CRISPR sequence leaders

To perform this analysis, 18 leader sequences were obtained in order to assess the evolutionary relationship among the CRISPR loci. For each CRISPR locus, there were 6 sequences from published genomes, 3 *Y. pestis* (Antiqua: NC_008150**,** KIM: NC_004088, CO92: NC_003143) and 3 *Y. pseudotuberculosis* (IP32953: NC_006155, PB1/+: NC_010634, YPIII: NC_010465). It is worth to mention that isolates of *Yersinia pseudotuberculosis* were used as ancestral representatives of *Y. pestis*. The first step was sequence alignment using MAFFT software [16]. It was used in local multiple alignment mode which is suitable for analysis of a set of sequences that may possess isolated motifs. Afterward, the alignment generated was trimmed using TrimAL [17] to select blocks of conserved regions. Then, in order to choose the evolutionary model which best fits with this alignment, eleven substitution models were tested including models with equal/unequal base frequencies, models with/without a proportion of invariable sites, and models with/without rate variation among sites. In the end, 88 models were tested using jModel Test [18], [19]. The trimmed alignment was used as input for the PhyML version 3.0. This is a simple, fast and accurate algorithm to estimate large phylogenies by maximum likelihood, which is a probabilistic method for evolutionary inference [20]. Finally, the tree was read in FigTree version 1.2.3 software (http://tree.bio.ed.ac.uk/software/figtree), which is a tree figure drawing tool. The leader sequence of the locus CRISPR 17 from *Methanocaldococcus jannaschii* was used as the outgroup because it showed the greatest conservation when compared to other loci within the same organism.

### Sequences deposited in GenBank/NCBI

The CRISPR sequences were submitted and published in the GenBank database under the following accession numbers: YPa (GI:406828460; GI:406828461; GI:406828462; GI:406828463; GI:406828464; GI:406828465; GI:406828466; GI:406828467; GI:406828468; GI:406828469; GI:406828470; GI:406828471; GI:406828472; GI:406828473) and YPb (GI:406828474; GI:406828475; GI:406828476; GI:406828477; GI:406828478; GI:406828479).

## Results

### The CRISPR loci structure in *Yesinia pestis*

The 3 CRISPR loci were identified for all *Y. pestis* strains (Table S1), except for Angola strain, which showed only the YPa locus, in addition to the degenerate repeat and the leader sequence of the YPb and YPc loci [4], [5]. For all strains, the direct repeats (DR) and degenerate repeats (DG) showed conservation to the analyzed loci.

**Figure 1:**
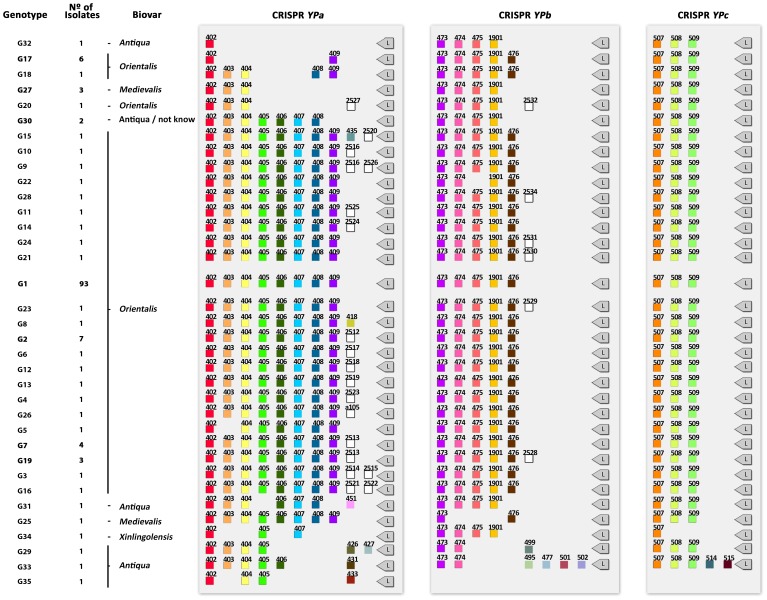
Groups of *Y. pestis* strains in accordance with the CRISPR spacer array of the three loci. Representation of spacer arrays distributed among the total number of identified genotypes: Colored boxes, spacers previously described [3], [4], [5]. White boxes, new spacers. L: leader sequence.

**Figure 2:**
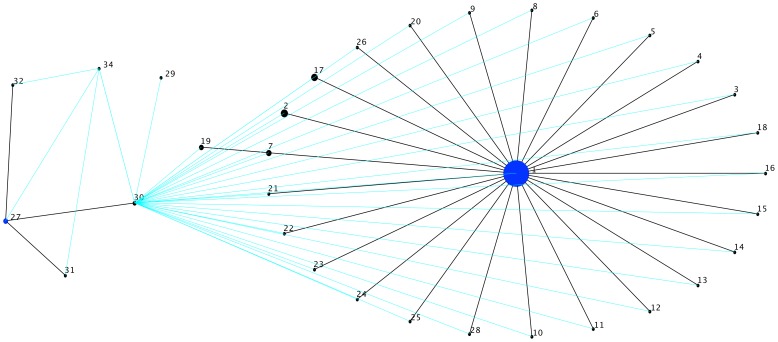
Genotype network of the *Yersinia pestis* strains. Main clonal complex composed of 26 genotypes and encompassing 135 isolates; Secondary clonal complex composed of six genotypes and distributed among nine isolates.

Identification of spacer sequences from CRISPR loci was conducted to evaluate the extent of genotypic diversity among the *Y. pestis* isolates. According to the sequence spacer arrays (presence or absence), 146 *Y. pestis* strains were grouped into 35 different genotypes (G1 – G35) (Fig. 1). The genotype G1 appeared as the most frequent (93 strains; 64%), followed by genotypes G2 (7 strains; 5%), G17 (6 strains; 4%), G7 (4 strains; 3%), G19 and G27 (3 strains; 2%), and G30 (2 strains; 1%). The remaining strains (28 strains; 19%) showed exclusive genotypes. The genotypes were clustered in 2 complexes (Fig. 2). In addition, two genotypes did not fit to any complex (G33, G35). The main clonal complex was defined with 26 genotypes distributed among 135 isolates. The genotype G1 was considered the founder genotype as it clustered to the largest number of single locus variant (SLV) genotypes (Fig. 2). The secondary clonal complex had 6 different genotypes with a total of 9 isolates, genotype 27 being the most frequent (3 isolates) and with the largest number of SLV’s (Fig. 2).

It is worthwhile to mention that all strains placed into the secondary clonal complex are from biovars other than the *orientalis* biovar. In addition, genotype G30 was indicated as a possible link or the closest genotype to the one which would be the link between both clonal complexes. Genotype G30 differs by the absence of two spacer sequences (409 and 476) present in genotype G1.

**Figure 3:**
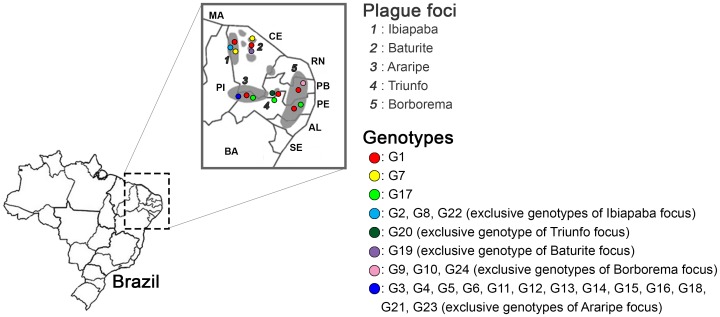
Genotype distribution and natural plague foci on the map of Brazil. Color circles are the 24 exclusives genotypes and letters are the plague foci from Brazil.

Insertion and deletion of spacer sequences into the CRISPR loci were identified in the analyzed strains. A total of 21 new spacers were found in the strains from plague foci of Brazil, 16 to the YPa and five to the YPb. No modifications were observed in the YPc locus of the Brazilian strains. Eleven spacers were similar to viral, plasmid and chromosomal sequences (Table 1). The new spacers were called Region-Specific-Spacers (RSS) and were grouped in the G2–G24 genotypes (Fig. 3). We observed spacer-specific plague foci which were distributed in all plague foci in Brazil (Fig. 3), unlike the situation with the G1 genotype.

**Table 1:** Characterization of the new spacers in *Yersinia pestis*.

Spacer	Sequence	Genic and Chromosomal Region	Product
2512	TTTTGCATTTGGATTCTCCTTGAATGCCTCACT	YPO2125	Phage Regulatory Protein
2513	CAGCCAGATAGCCGTTTTTCACAGTATTGATA	YPO2108	Hypothetical Phage Protein
2514	AGATCATGGGGCCGAGTTAGAACATCAAACAT	23S ribosomal RNA#	rRNA-23S ribosomal RNA
2515	ATAGGCATAGCACCGAGGCGGCGCGAAAACAG	YPO3683	Transcriptional Regulator
2516	AGGTGCAACAGGGACTTTAGGATAGAAAAGTCC	fosfolipaseD	pMT1 Plasmid Region
2517	ACTTAGGGACATTAGCTTGGGATGTGAAACAG	YPO3682	Transcriptional Regulator
2518	GTGTGGGTTTCGACATCCAACAACTGCCAAAT	YPO3722	Methyltransferase
2519	AAGCTAAAGGCCCGCCGTTTGTGGTGGTACCA	pMT1	pMT1 Plasmid Region
2520	ATGTAGTTCCCGCTGGAACTTGTCCATCCATA	YPO2108	Hypothetical Phage Protein
2521	CCTTTCCCAGTAGAGCTGAACCATCTTTATCA	YPO2109	Hypothetical Phage Protein
2522	GTTTGCTACCATCACCGCCAGTAGTGTATCCC	YPO1270	Protein ABC transporter
2523	CTTGACCCTCAAATTGAGTGTAAAGGGGTTTGG	YPO2093	Phage Protein
2524	GGTAAGCTCTGCATTTAACGCTGTTTCGACGG	y1062^+^	Transposase
2525	TGTTTCCACGTTGCAATTTTTCACCATGCTTA	pCD1	pCD1 Plasmid Region
2526	ATTGATGACTAGAATACACTAGTAAGTAATAAC	fosfolipaseD	pMT1 Plasmid Region
2527	GCTCTGCGTCACTCTCATTGAGCACTTTAACC	YPO2108	Hypothetical Phage Protein
2528	TGCCTTTTGCAGCCAGTCGCGCCACTCTTCGG	YPO2106	Phage Protein
2529	TGCGCCATTGGCGTTGGTTTTCAGGTATTCCAG	YPO2106	Phage Protein
2530	GAAAGAATAAGGATTTATAATTTATGACCACA	YPO2109; YPO2110*	Hypothetical Phage Protein
2531	ACCAAGCGGAACTGCCTCAAAAGCACCGGTTA	YPO2103	Phage Terminase
2532	GTGGCGATAAACTTAAGCTGGGTCAAGATTAT	YPO2095; YPO2096*	Hypothetical Phage Protein
2533	GCATAACCGCCGAGGACGCTGTTAAATACTTT	YPO2114	Hypothetical Phage Protein
2534	GAGAATCGTTGCAGGATAGTTTTCACCGCGT	YPO2109	Hypothetical Phage Protein

# 5 gene copies in the genome; + gene coding a transposable element, compared with the reference strain CO92 and verified by Blast. 20 gene copies; *Location between 2 genes in the reference strain CO92. b51 (localization: 2.381.302–2.381.333), similarity with regions of the YPO2109 (localization: 2.380.538–2.381.311) and YPO2110 (localization: 2.381.325–2.382.530) genes. b53 (localization: 2.370.526–2.370.557), similarity with regions of the YPO2095 (localization: 2.370.351–2.370.560) and YPO2096 (localization: 2.370.557–2.370.832).

Deletions were observed for 7 spacers in YPa locus (G5, G17, G18, G20, G27, G29, G30, G31, G32, G33, G34, G35) and for 3 spacers in the YPb (G20, G22, G25, G27, G29, G30, G31, G32, G33, G34) (Fig. 1). A high degree of diversity of spacers in the YPa locus was observed in the representatives of the Antiqua biovar. The same spacer arrays in YPb locus was found D182038 (G31), Nepal516 (G32) and Z176003 (G30) strains. Moreover, the Antiqua strain showed insertion and deletion in the YPa and YPb loci. Only the Pestoides F strains (G33) showed modifications in 3 loci (Fig. 1).

Medievalis biovar spacers/spacer arrays were the same in strains KIM, PKR684 and Harbin35 (G27) (Fig. 1).

We also observed that the representative of the Xilingolensis biovar (G34) showed exclusive spacer arrays in the YPa and YPc loci [3], [4], and the same arrays were present in the strains of the Antiqua and Medievalis biovars for the YPb locus (Fig. 1).

### Evolutionary relationship of CRISPR leader sequences

**Figure 4:**
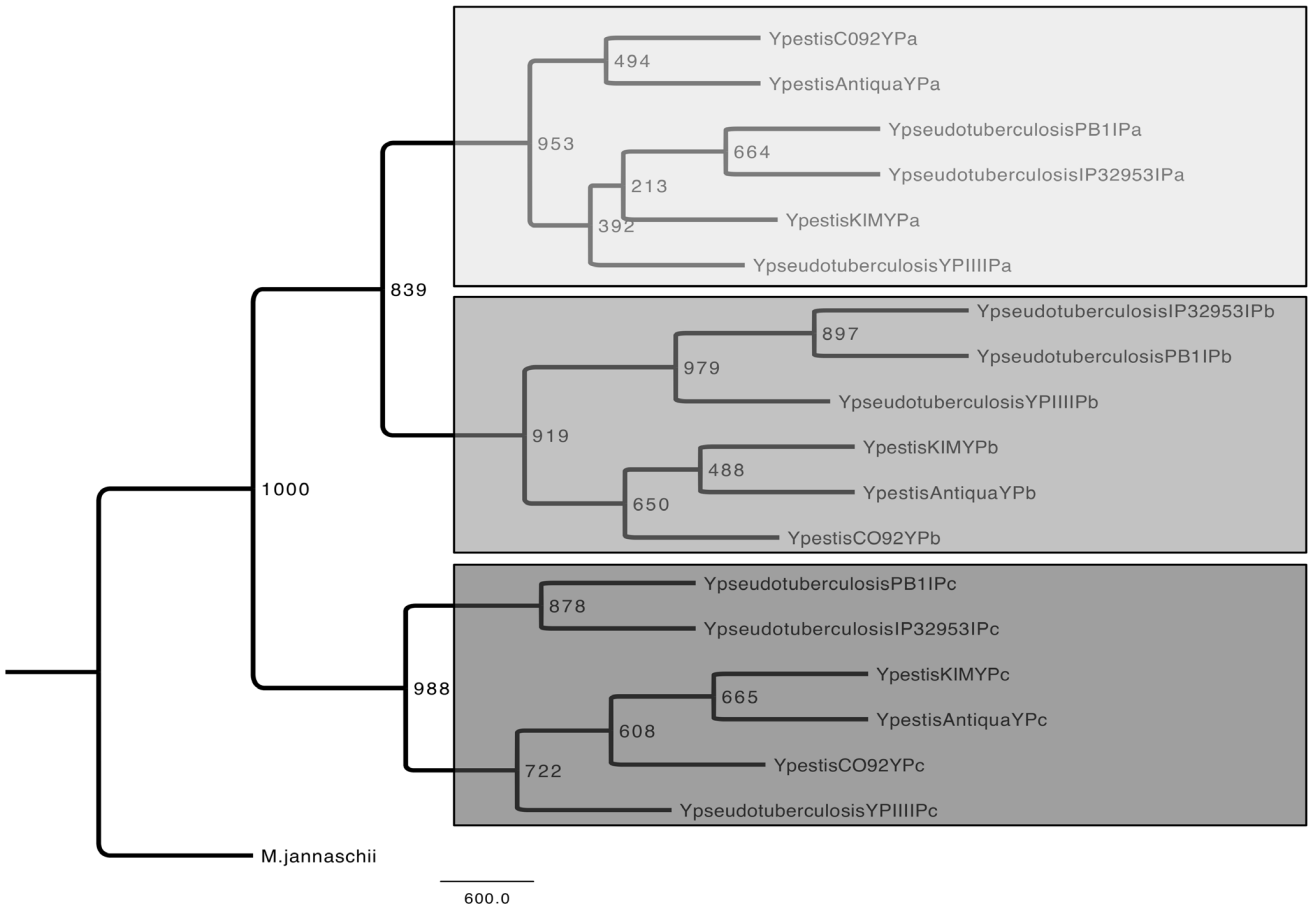
Tree generated by the alignment of 18 leader sequences of CRISPR loci of *Yersinia pestis* and *Yersinia pseudotuberculosis*. I, II, III – main groups: YPa, YPb and YPc loci of *Y. pestis* and *Y. pseudotuberculosis*. A - important branch in the emergence of the YPa and YPb loci; B - unique branch in the emergence of the YPc locus.

Using the leader sequences from the three distinct kinds of CRISPR loci analyzed in this study, a tree was built (Fig. 4). It is possible to notice that sequences, which belong to the same CRISPR locus clustered into three main groups (Fig. 4). Groups I and II seemed to be closer to each other since they share a common ancestral leader sequence more recent than the common ancestral leader sequence of the three groups. Groups I and II contain the leader sequences of the CRISPR loci YPa and YPb of *Y. pestis* and *Y. pseudotuberculosis*, and group III is made of leader sequences of the loci YPc (Fig. 4).

## Discussion

### CRISPR polymorphism in *Y. pestis*

The feature observed in the Angola strain (G35) reinforces its position as the most ancestral lineage of *Y. pestis* [3], [5]. This strain has the ability to ferment melibiose and rhamnose, a property associated with *Y. pseudotuberculosis* and *Y. pestis* subsp. microtus strains (Pestoides) [8]. Its chromosome is characterized by an intermediate genetic formation between *Y. pestis* and *Y. pseudotuberculosis* strains. Genotypic and phenotypic analyses suggested that the Angola strain belongs to one of the most ancient lineages of *Y. pestis* [21].

Given the natural dynamic of acquisition of new spacers observed in this study, we also conclude that this is the target polymorphic region for studies of subtyping of bacterial strains. No modifications was observed in the YPc locus in the isolates of *Y. pestis* from plague foci in Brazil, however a high degree of diversity was observed in the YPa and YPb loci, **similar** to **previous** work [3], [4], [5].

In accordance with the tree topology, the leader sequences of the YPc locus might have diverged earlier. Therefore, we suggest that the YPc locus leader sequences might be closer to the last common ancestral leader sequence of the CRISPR loci in the genus Yersinia. This result would suggest that the YPa and YPb loci are more closely related to each other than either of them to the YPc locus, confronting the hypothesis proposed by Pourcel et al. [3].

The majority of isolated plague foci from Brazil showed a spacer array identical to the reference strain, CO92. This spacer array has already been defined as the CRISPR for strains of the Orientalis biovar [4], [5].

We also identified insertions of focus-specific-spacers adjacent to the leader sequence and deletions of more internal spacers [4], [5]. Barrangou and Horvath [22] suggested that the deletions occur by homologous recombination between the CRISPR repeats. In addition, deletion of older spacers allows the conservation of spacers related to viruses circulating in the environment.

Our analysis confirmed that the CRISPR regions of the *Y. pestis* strains from plague foci in Brazil harbor spacers that can act against mobile genetic elements. The similarity found in spacers 2516, 2519, 2525, 2526 with typical *Y. pestis* plasmids (pMT1 and pCD1) may be due to possible integration of plasmid fragments into the CRISPR loci. One study showed that the CRISPR-Cas system is able to acquire spacers from plasmids present in the bacterial genome [23]. Future studies may clarify the information found in that study.

### Epidemiological study of *Y. pestis*

Our results suggest the G1 genotype as the founder, which could be responsible for spreading *Y. pestis* in these foci. This genotype showed the largest number of SLV genotypes in the complex. In this clonal complex, genotypes 7 was considered subgroup founder because each it has 3 SLV’s (G19) in the main clonal complex.

Our analysis suggests a possible intermediary genotype (G30) between the main and secondary clonal complex. The determination of this intermediate group is suggested by the absence of two spacers (409, 476) which were likely acquired by the G1 genotype when it increased its population size. In addition, the G30 genotype was found in two strains, one of which is of an unknown biovar (D106004: NC_017154). The main complex grouped all Brazilian strains with the Orientalis biovar, only two strains were foreign (G25, G26 and G28). Thus, the CRISPR locus was able to separate the strains of the Orientalis biovar. The secondary complex grouped the other biovars and showed the G27 genotype as a founder, as it shared the largest number of SLV’s.

The molecular data supported the historical evidence of the spread of an ancestral lineage, reinforcing the hypothesis of a single clonal entry into Brazil [10], [24]. The other genotypes showed distinct subpopulations of *Y. pestis* that demonstrated bacterial microevolution in these environments.

According to Zhou et al. [25] and Li et al. [26], adaptive microevolution promotes diversification of *Y. pestis* strains in major and secondary genotypes within the enzootic foci. The main genotypes play a crucial role in maintaining these plague foci, while secondary genotypes contribute to adaptation and a balanced interaction of the environment - host / reservoir - *Y. pestis*.

The genotypic variations found in this study are attributed to the following genetic events [4]: 1. random deletion of one or more spacers; 2. addition of new spacers in a polarized way near the leader sequence. Among the new spacers inserted, we identified Region-Specific-Spacers (RSS) in *Y. pestis* strains isolated from plague foci in Brazil. These RSS were located in specific areas of plague foci in the country.

Some new spacers are peculiar: 2512 (G2) was identified only in the Ibiapaba (CE) focus, between rodents (*Necromys lasiurus*, *Calomys callosus*) and humans, during the period of 1979 to 1986 (Table S1). The spacer 2513 (G7) was found in the Baturité (CE) focus, in the year 1978, and between a rodent (*Calomys callosus*) and humans. Curiously, it was also identified in an isolate from humans in 1979 in the Ibiapaba (CE) focus. These regions have territorial proximity (approximately 340 km). It is possible that the host moved among the foci (Fig. 3). The insertion of the spacer 2529 (G19) and 2513 (G19) were again identified after three and four years (1982, 1983) in the same focus region. The data from this study shows the applicability of CRISPR loci analysis in temporal and geographical study in isolates of *Y. pestis* from plague foci in Brazil.

The remaining spacers were related to a specific isolate, but with exclusive features: six were found in humans (2514, 2515, 2523, 2527, 2530, 2533), four in fleas *Polygenis bohlsi jordani* (2517, 2518, 2524, 2525), ten in rodents: *Rattus rattus* (418, 2516, 2519, 2526), *Necromys lasiurus* (435, 2520, 2521, 2522, 2532) and *Orysomys subflavus* (2531). These spacers can be considered markers to track plague in these host-reservoirs.

## Conclusion

This study identified new spacers and provided significant data on the diversity of CRISPR loci. Moreover, it allowed observing a microevolutionary process in a group of *Y. pestis* isolated from Brazil. The data from this study shows the applicability of CRISPR loci analysis in temporal and geographical study in isolates of *Y. pestis* from plague foci in Brazil. We also identified a founder group responsible for the spreading of the bacteria in the plague foci in Brazil. In addition, the homogeneity of the strains is possible evidence for a single entry of *Y. pestis* into Brazil. Further studies are needed to clarify some functional and evolutionary questions of the CRISPR loci in the *Yersinia pestis* species.

## Supporting Information

Table S1 **Characteristics of the** ***Yesinia pestis*** **strains studied.**
(DOC)
