# Supplementary material for: Dynamics of CRISPR Loci in Microevolutionary Process of Yersinia pestis Strains
Source: PLoS One. 2014 Sep 29;9(9):e108353. doi: 10.1371/journal.pone.0108353 (PMC4180756; doi:10.1371/journal.pone.0108353)
Supplement: Table S1 — Characteristics of the Yesinia pestis strains studied. (DOC) [file pone.0108353.s001.doc]

**Table S1**

**Characteristics of the *Yesinia pestis* strains studied**.

Strains of plague foci of Brazil

| **Strain** | **Year of Isolation** | **Plague Focus** | **Source of Isolation** | **Municipality/Country** | **Farm** | **Biovar** |
| --- | --- | --- | --- | --- | --- | --- |
| P. CE 01 | 1978 | Ibiapaba | Human | CE | s/i | *Orientalis* |
| P. CE 03 | 1979 | Ibiapaba | *Necromys lasiurus* | Ipu-CE | s/i | *Orientalis* |
| P. CE 09 | 1980 | Ibiapaba | *Necromys lasiurus* | CE | s/i | *Orientalis* |
| P. CE 11 | 1982 | Ibiapaba | *Necromys lasiurus* | CE | s/i | *Orientalis* |
| P. CE 13 | 1982 | Baturité | *Necromys lasiurus* | CE | s/i | *Orientalis* |
| P. CE 14 | 1982 | Ibiapaba | *Pulex irritans* | CE | s/i | *Orientalis* |
| P. CE 18 | 1982 | Ibiapaba | *Necromys lasiurus* | CE | s/i | *Orientalis* |
| P. CE 19 | 1982 | Ibiapaba | *Necromys lasiurus* | CE | s/i | *Orientalis* |
| P. CE 21 | 1982 | Ibiapaba | Human | CE | s/i | *Orientalis* |
| P. CE 24 | 1982 | Baturité | *Rattus rattus* | CE | s/i | *Orientalis* |
| P.CE 882 | 1997 | Ibiapaba | Human | Ipu-CE | s/i | *Orientalis* |
| P. Exu 12 | 1966 | Araripe | *Orysomys eliurus* | Exu-PE | Serra Manoel Severino | *Orientalis* |
| P. Exu 19 | 1967 | Araripe | *Orysomys subflavus* | Exu-PE | Alto | *Orientalis* |
| P. Exu 241 | 1968 | Araripe | *Rattus rattus (Rra)* | Exu-PE | Gadelha | *Orientalis* |
| P. Exu 365 | 1970 | Araripe | *Necromys lasiurus* | Exu-PE | Espalhado | *Orientalis* |
| P. Exu 374 | 1970 | Araripe | *Necromys lasiurus* | Exu-PE | Cacimbas | *Orientalis* |
| P.Exu 375 | 1970 | Araripe | *Necromys lasiurus* | Exu-PE | Espalhado | *Orientalis* |
| P. Exu 377 | 1970 | Araripe | *Necromys lasiurus* | Exu-PE | Espalhado | *Orientalis* |
| P. Exu 430 | 1971 | Araripe | *Orysomys subflavus* | Exu-PE | Pamonha | *Orientalis* |
| P. Exu 509 | 1971 | Ibiapaba | Human | Guaraciaba do Norte-CE | Timbauba | *Orientalis* |
| P. Exu 527 | 1971 | Ibiapaba | Human | Guaraciaba do Norte-CE | Burití Apuá | *Orientalis* |
| P. Exu 538 | 1971 | Ibiapaba | Human | Ipu-CE | Mato Grosso | *Orientalis* |
| P. Exu 540 | 1971 | Ibiapaba | Human | Ipueiras-CE | Povoado América | *Orientalis* |
| P. Exu 542 | 1971 | Ibiapaba | Human | Guaraciaba do Norte-CE | Lagoa do Firmino | *Orientalis* |
| P. Exu 554 | 1971 | Ibiapaba | Human | São Benedito-CE | Pimenteira | *Orientalis* |
| P. Exu 556 | 1972 | Ibiapaba | Human | Guaraciaba do Norte-CE | São Felix | *Orientalis* |
| P. Exu 558 | 1972 | Ibiapaba | Human | Ipu-CE | Macambira | *Orientalis* |
| P. Exu 617 | 1974 | Araripe | *Polygenis b. jordani* | Exu-PE | Se. Pau Casado | *Orientalis* |
| P. Exu 715 | 1974 | Araripe | Human | Exu-PE | Alto do Ferreira | *Orientalis* |
| P. Exu 718 | 1974 | Araripe | *Necromys lasiurus* | Exu-PE | São Luiz | *Orientalis* |
| P. Exu 720 | 1974 | Araripe | *Polygenis b. jordani* | Exu-PE | Barro | *Orientalis* |
| P. Exu 738 | 1975 | Araripe | Human | araripina-PE | Cansanção | *Orientalis* |
| P. Exu 791 | 1978 | Triunfo | *Necromys lasiurus* | Triunfo-PE | Barbalho | *Orientalis* |
| P. Exu 792 | 1978 | Ibiapaba | Human | Ipu-CE | Cabeça Dantas | *Orientalis* |
| P. Exu 798 | 1978 | Triunfo | *Necromys lasiurus* | Triunfo-PE | S. José dos Pilotos | *Orientalis* |
| P. Exu 799 | 1978 | s/i | Rodent (s/i) | Serrinha-BA | Flores | *Orientalis* |
| P. Exu 801 | 1978 | Ibiapaba | Human | Ipu-CE | Tabocal | *Orientalis* |
| P. Exu 822 | 1979 | Triunfo | *Orysomys subflavus* | Triunfo-PE | Lagoa do Almeida | *Orientalis* |
| P. Exu 835 | 1979 | Ibiapaba | *Necromys lasiurus* | Ipu-CE | Taboca | *Orientalis* |
| P. Exu 861 | s/i | Ibiapaba | *Necromys lasiurus* | Ipueiras-CE | Grossos | *Orientalis* |
| P. PB 862 | 1986 | Borborema | Human | Solâne-PB | Pedra d'Água | *Orientalis* |
| P. PB 864 | 1986 | Borborema | *Rattus rattus (Rrf)* | Solâne-PB | Pedra d'Água | *Orientalis* |
| P. PB 867 | 1986 | Borborema | *Necromys lasiurus* | Solâne-PB | Lagoa do Serrote | *Orientalis* |
| P. PB 868 | 1986 | Borborema | *Necromys lasiurus* | Solâne-PB | Cinco Estrelas | *Orientalis* |
| P. PB 869 | 1986 | Borborema | *Holochilus sciureus* | Solâne-PB | Pedra d'Água | *Orientalis* |
| P. PB 870 | 1986 | Borborema | *Necromys lasiurus* | Solâne-PB | Pedra d'Água | *Orientalis* |
| P. PB 871 | 1986 | Borborema | *Necromys lasiurus* | Solâne-PB | Pedra d'Água | *Orientalis* |
| P. PB 872 | 1986 | Borborema | *Necromys lasiurus* | Solâne-PB | Cinco Lagoas | *Orientalis* |
| P. PB 873 | 1986 | Borborema | *Orysomys subflavus* | Solâne-PB | Cinco Lagoas | *Orientalis* |
| P. PB 874 | 1986 | Borborema | *Rattus rattus(Rrf)* | Solâne-PB | Bacalhau | *Orientalis* |
| P. PB 877 | 1986 | Borborema | *Rattus rattus(Rrf)* | Solâne-PB | Cinco Lagoas | *Orientalis* |
| P. PB 878 | 1986 | Borborema | *Orysomys subflavus* | Solâne-PB | Bacalhau | *Orientalis* |
| P. PB 879 | 1986 | Borborema | *Orysomys subflavus* | Solâne-PB | Pedra d'Água | *Orientalis* |
| P. PB 880 | 1986 | Borborema | *Orysomys subflavus* | Solâne-PB | Bacalhau | *Orientalis* |
| P. PB 881 | 1986 | Borborema | Human | Remígio-PB | Serrinha | *Orientalis* |
| P. Exu 30 | 1967 | Araripe | *Polygenis b. jordani* | Exu-PE | Alagoinha | *Orientalis* |
| P. Exu 31 | 1967 | Araripe | *Necromys lasiurus* | Exu-PE | Alagoinha | *Orientalis* |
| P. Exu 39 | 1967 | Araripe | *Necromys lasiurus* | Exu-PE | Alagoinha | *Orientalis* |
| P. Exu 42 | 1967 | Araripe | *Necromys lasiurus* | Exu-PE | Alagoinha | *Orientalis* |
| P. Exu 42 | 1967 | Araripe | *Necromys lasiurus* | Exu-PE | Alagoinha | *Orientalis* |
| P. Exu 47 | 1967 | Araripe | *Necromys lasiurus* | Exu-PE | Alagoinha | *Orientalis* |
| P. Exu 48 | 1967 | Araripe | *Necromys lasiurus* | Exu-PE | Alagoinha | *Orientalis* |
| P. Exu 53 | 1967 | Araripe | *Necromys lasiurus* | Exu-PE | Alagoinha | *Orientalis* |
| P. Exu 57 | 1967 | Araripe | *Necromys lasiurus* | Exu-PE | Alagoinha | *Orientalis* |
| P. Exu 57 | 1967 | Araripe | *Necromys lasiurus* | Exu-PE | Alagoinha | *Orientalis* |
| P. Exu 60 | 1967 | Araripe | *Rattus rattus(Rrf)* | Exu-PE | Alagoinha | *Orientalis* |
| P. Exu 60 | 1967 | Araripe | *Rattus rattus(Rrf)* | Exu-PE | Alagoinha | *Orientalis* |
| P. Exu 74 | 1967 | Araripe | *Xenopsylla cheopsi* | Exu-PE | Alagoinha | *Orientalis* |
| P. Exu 15 | 1966 | Araripe | Human | Exu-PE | Retiro | *Orientalis* |
| P. Exu 185 | 1967 | Borborema | Human | Caruaru-PE | Lagoa do Anjo | *Orientalis* |
| P. Exu 246 | 1968 | Araripe | Human | Araripina-PE | Cascavel | *Orientalis* |
| P. Exu 276 | 1968 | Araripe | Human | Bodocó-PE | Queimada | *Orientalis* |
| P. Exu 292 | 1968 | Triunfo | Human | São José do Belmonte-PE | Mata Redonda | *Orientalis* |
| P. Exu 312 | 1969 | Araripe | Human | Exu-PE | Badreci | *Orientalis* |
| P. Exu 363 | 1970 | Araripe | *Necromys lasiurus* | Exu-PE | Cacimbas | *Orientalis* |
| P. Exu 373 | 1970 | Araripe | *Polygenis b. jordani* | Exu-PE | Cacimbas | *Orientalis* |
| P. Exu 384 | 1970 | Araripe | *Polygenis b. jordani* | Exu-PE | Oriente | *Orientalis* |
| P. Exu 459 | 1971 | Araripe | *Polygenis b. jordani* | Exu-PE | Aracajú | *Orientalis* |
| P. Exu 602 | 1974 | Araripe | *Necromys lasiurus* | Exu-PE | Serra Pau Casado | *Orientalis* |
| P. Exu 623 | 1974 | Araripe | Rodent *(gsw)* | Exu-PE | Serra Pau Casado | *Orientalis* |
| P. Exu 769 | 1975 | Araripe | Human | Ipubi-PE | Cacimbinha | *Orientalis* |
| P. Exu 771 | 1975 | Araripe | Human | Araripina-PE | Serra Antônio Domingos | *Orientalis* |
| P. Exu 778 | 1975 | Araripe | Human | Araripina-PE | Cavaco | *Orientalis* |
| P. Exu 781 | 1975 | Araripe | Human | Granito-PE | Catumbi | *Orientalis* |
| P. Exu 788 | 1977 | Araripe | *Polygenis b. jordani* | Araripina-PE | Alto Alegre | *Orientalis* |
| P. Exu 793 | 1978 | Triunfo | *Necromys lasiurus* | Triunfo-PE | Jericó | *Orientalis* |
| P. Exu 813 | 1979 | Triunfo | *Necromys lasiurus* | Triunfo-PE | Salgada | *Orientalis* |
| P. Exu 825 | 1979 | Borborema | *Polygenis b. jordani* | Tacaimbó-PE | Malhada do Juá | *Orientalis* |
| P. Exu 827 | 1979 | Borborema | *Polygenis b. jordani* | São Caetano-PE | Campo Alegre | *Orientalis* |
| P. CE 04 | 1979 | Ibiapaba | *Necromys lasiurus* | CE | s/i | *Orientalis* |
| P. CE 05 | 1979 | Ibiapaba | *Necromys lasiurus* | Guaraciaba do Norte-CE | s/i | *Orientalis* |
| P. CE 06 | 1979 | Ibiapaba | Rodent (s/i) | CE | s/i | *Orientalis* |
| P. CE 07 | 1979 | Ibiapaba | *Necromys lasiurus* | CE | s/i | *Orientalis* |
| P. CE 30 | 1986 | Ibiapaba | *Calomys callosus* | CE | s/i | *Orientalis* |
| P. Exu 789 | s/i | Ibiapaba | Human | Guaraciaba do Norte-CE | São Felix | *Orientalis* |
| P. Exu 842 | s/i | Ibiapaba | *Necromys lasiurus* | Guaraciaba do Norte-CE | São Felix | *Orientalis* |
| P. Exu 184 | 1967 | Araripe | Human | Exu-PE | Serra Jatobá | *Orientalis* |
| P. Exu 334 | 1969 | Araripe | Human | Exu-PE | Gambá | *Orientalis* |
| P. Exu 340 | 1969 | Araripe | Human | Ipubi-PE | Se. Anastácio Barbosa | *Orientalis* |
| P. Exu 787 | 1976 | Araripe | *Polygenis b. jordani* | Exu-PE |  | *Orientalis* |
| P.Exu 795 | 1978 | Baturité | Human | Aratuba-CE | Serrinha | *Orientalis* |
| P. Exu 796 | 1978 | Baturité | Human | Aratuba-CE | Serrinha | *Orientalis* |
| P. Exu 806 | 1978 | Baturité | *Calomys callosus* | Aratuba-CE | Fernandes | *Orientalis* |
| P. Exu 809 | 1979 | Ibiapaba | Human | Ipu-CE | Macaco | *Orientalis* |
| P. Exu 797 | 1978 | Ibiapaba | *Rattus rattus* | Ipu-CE | Almas | *Orientalis* |
| P. PB 866 | 1986 | Borborema | *Rattus rattus(Rrf)* | Solâne-PB | Valério | *Orientalis* |
| P. PB 876 | 1986 | Borborema | *Rattus rattus(Rrf)* | Solâne-PB | Cinco Lagoas | *Orientalis* |
| P. Exu 36 | 1967 | Araripe | *Polygenis b. jordani* | Exu-PE | Alagoinha | *Orientalis* |
| P. Exu 44 | 1967 | Araripe | *Polygenis b. jordani* | Exu-PE | Alagoinha | *Orientalis* |
| P. Exu 54 | 1967 | Araripe | *Rattus rattus(Rra)* | Exu-PE | Alagoinha | *Orientalis* |
| P. Exu 59 | 1967 | Araripe | *Polygenis b. jordani* | Exu-PE | Alagoinha | *Orientalis* |
| P. Exu 61 | 1967 | Araripe | *Necromys lasiurus* | Exu-PE | Alagoinha | *Orientalis* |
| P. Exu 67 | 1967 | Araripe | *Necromys lasiurus* | Exu-PE | Alagoinha | *Orientalis* |
| P. Exu 17 | 1966 | Triunfo | Human | Triunfo-PE | Santa Clara | *Orientalis* |
| P. Exu 248 | 1968 | Triunfo | Human | São José do Belmonte-PE | Baixa verde | *Orientalis* |
| P. Exu 249 | 1968 | Triunfo | Human | São José do Belmonte-PE | Bananeira 2º | *Orientalis* |
| P. Exu 351 | 1969 | Triunfo | Human | Triunfo-PE | Batinga | *Orientalis* |
| P. Exu 674 | 1974 | Araripe | Human | Exu-PE | Brejo Santo Inácio | *Orientalis* |
| P. Exu 832 | 1979 | Borborema | *Polygenis b. jordani* | Tacaimbó-PE | Paulista | *Orientalis* |
| P. Exu 387 | 1970 | Araripe | *Necromys lasiurus* | Exu-PE | Oriente | *Orientalis* |
| P.CE 17 | 1982 | Baturité | *Necromys lasiurus* | Aratuba-CE | s/i | *Orientalis* |
| P.CE 25 | s/i | s/i | *s/i* | *s/i* | s/i | *Orientalis* |
| P. CE 28 | 1983 | Baturité | Rodent (s/i)(nl ou gsw) | Mulungu-CE | s/i | *Orientalis* |
| P. Exu 16 | 1966 | Triunfo | Human | São José do Belmonte-PE | Minador | *Orientalis* |
| P. Exu 03 | 1966 | Araripe | *Orysomys subflavus* | Exu-PE | Serra Mundo Novo | *Orientalis* |
| P. Exu 803 | 1978 | Ibiapaba | Human | São Benedito-CE | Carnaúba | *Orientalis* |
| P. Exu 845 | 1980 | Araripe | Human | Ipubi-PE | Baixas | *Orientalis* |
| P. PB 865 | 1986 | Borborema | *Necromys lasiurus* | Solâne-PB | Cinco Lagoas | *Orientalis* |
|  |  |  |  |  |  |  |

Strains of others plague foci

| **Strain** | **Year of Isolation** | **Plague Focus** | **Source of Isolation** | **Biovar** |  |  |
| --- | --- | --- | --- | --- | --- | --- |
| PKOL | s/i | Curdistão | Human | *Orientalis* |  |  |
| ALEXANDER | s/i | EUA | Human | *Orientalis* |  |  |
| KIM | s/i | Irã | Human | *Medievalis* |  |  |
| PKR684 | s/i | Irã | Rodent | *Medievalis* |  |  |
| PERU | 1995 | Peru | Human | *Orientalis* |  |  |
| PB8 | s/i | Birmânia | s/i | *Orientalis* |  |  |
| PBM5 | s/i | Birmânia | s/i | *Orientalis* |  |  |
| EV76 | 1926 | Madagascar | Human | *Orientalis* |  |  |
| A1122 | s/i | s/i | s/i | *Orientalis* |  |  |
| Angola | 1985 | s/i | s/i | *Antiqua* |  |  |
| Antiqua | 1965 | RDCongo | Human | *Antiqua* |  |  |
| D106004 | 2006 | Yulong County | Rodent | *s/i* |  |  |
| D182038 | 1982 | Yunnan | Rodent | *Antiqua* |  |  |
| Nepal516 | 1967 | Nepal | Human | *Antiqua* |  |  |
| Pestoide F | s/i | Russia | Rodent | *Antiqua* |  |  |
| Z176003 | 1976 | Tibet | Rodent | *Antiqua* |  |  |
| Harbin 35 | s/i | China | Human | *Medievalis* |  |  |
| 91001 | s/i | China | s/i | *Xilingolensis* |  |  |
